# The Relationship Between Developmental Dysplasia of the Hip (DDH) and Congenital Talipes Equinovarus (CTEV)—A Retrospective Case Series

**DOI:** 10.1111/jpc.70089

**Published:** 2025-05-19

**Authors:** Andrew Gorrie, Natalie Tannos, Dean Morris, Andrew Leicester, Jeffrey S. Ling, Arnold Suzuki, Samya Lakis

**Affiliations:** ^1^ School of Clinical Medicine University of New South Wales Kensington New South Wales Australia; ^2^ Physiotherapy Department Sydney Children’s Hospital Randwick New South Wales Australia; ^3^ Orthopaedics Department Sydney Children’s Hospital Randwick New South Wales Australia; ^4^ Graduate School of Medicine University of Wollongong Wollongong New South Wales Australia

**Keywords:** congenital talipes equinovarus, CTEV, DDH, developmental dysplasia of the hip, paediatrics

## Abstract

**Backgrounds:**

There is no consensus if a relationship exists between idiopathic congenital talipes equinovarus (CTEV) and developmental dysplasia of the hip (DDH). Our research aim was to provide a contemporary Australian population statistical relationship between DDH and idiopathic CTEV, compared to published data.

**Methods:**

We conducted a retrospective data analysis of a continuous sample of infants with idiopathic CTEV, who attended a Ponseti Clinic at Sydney Children's Hospital, born between 2010 and 2019. The prevalence of DDH requiring treatment was diagnosed utilising screening ultrasonography and compared to the most valid population data from Australia. The relative risk compared to this data was also determined.

**Results:**

There were 250 subjects who met the inclusion criteria and had complete datasets for analysis. The prevalence of DDH in our idiopathic CTEV study population was 52.9 (95% CI 27.6–90.5) per 1000. This represents a higher prevalence rate than Western Australian and South Australian datasets, with 9.5 (95% CI 8.9–10.1) and 5.0 (95% CI 4.6–5.5) per 1000, respectively. The relative risk of DDH in our idiopathic CTEV study population was 5.59 (95% CI 3.21–9.73, *p* < 0.0001) and 10.50 (95% CI 6.01–18.34, *p* < 0.0001), compared to Western Australian and South Australian population datasets, respectively.

**Conclusion:**

Our study findings support a positive correlation between idiopathic CTEV and DDH. The relative risk of DDH in the idiopathic CTEV population is 5–10 times higher than the general Australian population. When selective hip ultrasound screening is used, we believe idiopathic CTEV should be considered a risk factor for DDH.

## 1 Introduction

Developmental dysplasia of the hips (DDH), colloquially known as ‘clicky hips’, occurs when the head of the femur (ball) does not fit securely in the pelvic acetabulum (socket). DDH is a spectrum of disease encompassing frank dislocation of the hip, varying degrees of hip subluxation and instability, through to mild acetabular or femoral dysplasia, when parameters are outside the normative range. The gold standard diagnostic tool for DDH in newborns is hip ultrasonography. DDH is most often present at birth but can also develop in the first year of a child's life. The widely accepted risk factors at birth for DDH include: female, first born children, breech presentation, family history of DDH [1] and oligohydramnios [2]. It is also accepted that postnatal traditional swaddling is a risk factor for DDH [3]. The increased risk for DDH is associated with other intrauterine packaging deformities, including congenital muscular torticollis [4] and foot deformities [5].

Congenital structural talipes equinovarus (CTEV), also known as clubfoot, is a rigid foot deformity involving cavus, adductus, varus and equinus. The deformity is usually detected on antenatal ultrasound, but the diagnosis is confirmed on clinical examination at birth, assessed with midfoot and hindfoot Pirani scores. The Ponseti Method of treatment for CTEV is the most effective and accepted worldwide [6]. It involves neonatal serial casting, an Achilles tenotomy surgery in 90% of cases, and ‘boots and bar’ bracing during infancy. CTEV occurs in isolation (idiopathic), or in combination with other conditions (teratologic/syndromic) including arthrogryposis and spina bifida. The environmental and genetic causes of CTEV are not fully understood, but genetics seem to play a significant role [7].

Delayed diagnosis and initiation of treatment in DDH results in long‐term complications including abnormal formation of the hip joint, osteoarthritis and morbidity [8]. Late detection also increases the short‐term costs of treatment sevenfold [9], with potential need for greater invasiveness of treatment including surgery and casting as an infant through to femoral and acetabular osteotomies. Late detection in Australia is a trigger for potential medicolegal costs, in addition to higher health care costs associated with these outcomes.

Despite research on the relationship between DDH and CTEV, there is no consensus on whether a correlation exists. A 2023 British consensus study could not reach consensus on whether CTEV should be considered a risk factor for DDH [10]. A systematic review and meta‐analysis, published in 2015, determined a pooled prevalence of DDH in 4.1% of children with idiopathic CTEV, which they determined was similar to the general population [11]. The meta‐analysis included study populations between 24 and 672 subjects with CTEV and was heterogenous in design. The systematic review recommended that future higher quality studies were required to improve certainty. More recently, multiple studies have reported a positive correlation between DDH and CTEV; however, with low study numbers between 10 and 270 subjects affected by CTEV [2, 5, 12].

Our research aim was to provide a contemporary Australian population statistical relationship between DDH and idiopathic CTEV. This was performed by determining the prevalence of DDH in a continuous cohort of subjects with CTEV attending a specialist children's hospital service in Australia, compared to published general population data.

## 2 Materials and Methods

The study took place at Sydney Children's Hospital (SCH), a specialist children's hospital located in New South Wales (NSW), Australia. Since 2009, patients with CTEV referred to the hospital have been triaged to a physiotherapist and orthotist‐led Ponseti Clinic. Patients commence serial casting with the therapists and then see an orthopaedic surgeon for the Achilles tenotomy. Diagnosis of CTEV is made by a physiotherapist and confirmed by the orthopaedic surgeon. All attending children are routinely referred for a bilateral hip ultrasound to screen for DDH, to be completed between 6 and 8 weeks of age. An orthopaedic surgeon reviews the ultrasounds and diagnoses hips as normal, immature or dysplastic. The clinic is one of three Ponseti Clinics in NSW, also accepting external referrals from the Australian Capital Territory (ACT).

The researchers conducted a retrospective review of medical records. The study population was determined from a prospectively completed, continuous clinical database of patients attending the SCH, Ponseti Clinic. Two members of the research team (A.G., D.M.) collated the data from the clinical database to a research database for the purposes of the study. The presence of sex, breech presentation and torticollis was included in the data analysis. Being first born, family history of DDH, and postnatal swaddling were not recorded as they were not reliably available in the database. Hospital medical records were reviewed by the research team to complete missing data.

Subjects were included if they were born between 1 January 2010 and 31 December 2019; attended SCH for their initial casting block, and casting occurred before the age of 6 months. Subjects were excluded if they were predominantly managed externally as routine hip ultrasound was not reliably performed or recorded. Subjects with teratologic/syndromic CTEV, for example, arthrogryposis, spina bifida, were excluded.

The research team (D.M., S.L.) independently reviewed the medical imaging of subjects diagnosed with DDH on ultrasound. Hips with true dysplasia requiring treatment were defined as DDH, whereas immature or borderline hips which were monitored but not treated were defined as normal.

Statistical analysis was completed with the hospital statistician. The prevalence of DDH in the CTEV study population was determined, with 95% confidence intervals. Comparison was made to whole population datasets, relying on mandatory reporting of DDH by medical practitioners from Western Australia (WA) [13] and South Australia (SA) [14], as NSW does not have mandatory reporting. The research team analysed the published data from WA and SA to best represent the same period as the study population, 2010–2012 and 2012–2016, respectively. The relative risk was determined by comparison of this study data with the same datasets.

## 3 Results

**FIGURE 1:**
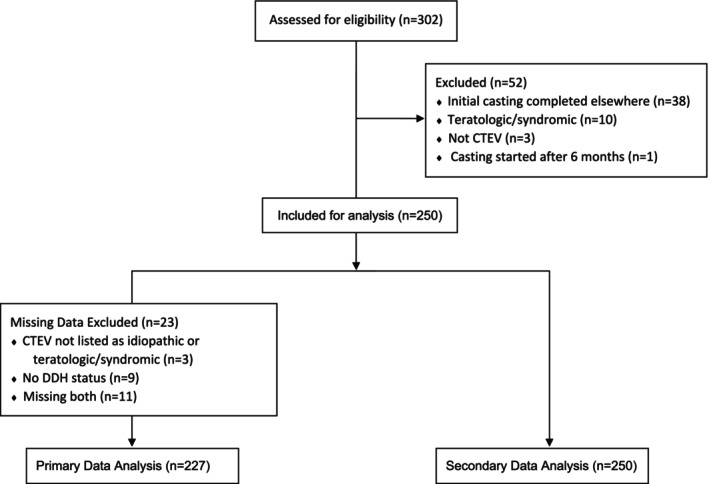
Inclusion/exclusion flow diagram.

There were 302 subjects in the clinical database born between 1 January 2010 and 31 December 2019. Fifty‐two were excluded, and 23 were unable to be analysed due to missing data (Figure 1). Two hundred and twenty‐seven subjects were included in the primary analysis; their demographics are presented in Table 1. All had midfoot and hindfoot Pirani scores, with the lowest recorded Pirani score being three.

**TABLE 1:** Demographics.

	Total (227)	Percent
Male	161	71
Female	66	29
Left CTEV	48	21
Right CTEV	79	35
Bilateral CTEV	100	44

There were 215 subjects in the normal hip group; 210 with a normal hip ultrasound and five with borderline hips/immaturity on ultrasound (Table 2). There were 12 with DDH requiring treatment (Table S1).

**TABLE 2:** DDH diagnoses by total population, sex and side/bilaterality CTEV.

	Normal hips	DDH	Percent
Total	215	12	5.29
Male	155	6	3.73
Female	60	6	9.09
Left CTEV	45	3	6.25
Right CTEV	76	3	3.80
Bilateral CTEV	94	6	6.00

Comparison of the prevalence rate is presented in Table 3. The prevalence of DDH in our study population with CTEV is 52.9 (95% CI 27.6–90.5) per 1000. This represents a higher prevalence rate than the WA dataset 2010–2012 of 9.5 (95% CI 8.9–10.1) per 1000 and SA dataset 2012–2016 of 5.0 (95% CI 4.6–5.5) per 1000. Comparison of the relative risk is presented in Table 4. The relative risk of DDH in our study population with CTEV is 5.59 (95% CI 3.21–9.73, *p* < 0.0001) and 10.50 (95% CI 6.01–18.34, *p* < 0.0001) compared to the WA and SA datasets, respectively.

**TABLE 3:** DDH prevalence rate of idiopathic CTEV population and normative data sets.

	Study population	DDH	Prevalence per 1000 (95% CI)
CTEV SCH 2010–2019	227	12	52.9 (27.6–90.5)
WA 2010–2012	97 318	920	9.5 (8.9–10.1)
SA 2012–2016	101 902	513	5.0 (4.6–5.5)

Abbreviations: SA, South Australia general population; SCH, Sydney Children's Hospital; WA, Western Australia general population.

**TABLE 4:** Relative risk of DDH in the idiopathic CTEV population compared to whole population cohort.

	Relative risk (95% CI)	*p*
Compared to WA 2010–2012	5.59 (3.21–9.73)	< 0.0001
Compared to SA 2012–2016	10.50 (6.01–18.34)	< 0.0001

Abbreviations: SA, South Australia general population; WA, Western Australia general population.

Secondary analysis was completed on 250 subjects with the assumption that the missing dataset had idiopathic CTEV and did not have DDH, to minimise positive skew. The prevalence rate was 48.0 (95% CI 25.0–82.3) per 1000. The relative risk under these conditions is 5.08 (95% CI 2.91–8.85, *p* < 0.0001) and 9.53 (95% CI 5.45–16.67, *p* < 0.0001), compared to the WA and SA population datasets, respectively.

## 4 Discussion

The results support a positive correlation between CTEV and DDH. There is strong evidence this prevalence of 52.9 (95% CI 27.6–90.5) per 1000 substantially differs from the general population. This is comparable to other positional foot deformities recommended to receive selective hip ultrasound screening: congenital talipes calcaneovalgus 65 per 1000, and metatarsus adductus 40 per 1000 [15]. There is an increased relative risk of children with CTEV having DDH; between 5.59 (95% CI 3.21–9.73) and 10.50 (95% CI 6.01–18.34). This risk is comparable to other risk factors that receive selective hip ultrasound screening: breech presentation 5.03 (95% CI 4.51–5.60) [11] and positive family history of DDH 12 [16].

These Australian results are consistent with recent overseas studies exploring the relationship between DDH and idiopathic CTEV. In a 2020 Norwegian study, six of the 170 subjects with CTEV, or 3.5%, were diagnosed with DDH using ultrasonography [5]. In a 2022 Chinese study examining 204 subjects with pathological DDH, 11 had CTEV, or 5.4% [2]. In addition to two subjects who presented with other foot deformities, they determined the relative risk of infants with foot deformities having DDH to be 13.00 (95% CI 3.32–51.21) [2]. In a 2017 Canadian study of 1469 infants with DDH, 42 were also diagnosed with CTEV, or 2.9% [12]. This represented a relative risk of 10.34 (95% CI 7.60–14.05) [12]. While a limitation in our study is the small CTEV sample relative to the comparison datasets, the 227 participants with CTEV in our study are relatively large compared to other studies.

Ultrasonography is considered the gold standard diagnostic tool for DDH in newborns, variably used from universal screening to a supplemental tool to clinical assessment and treatment, through to selective ultrasound screening in the setting of identifiable risk factors. Evidence suggests universal screening results in a significant increase in children treated with hip abduction bracing but does not decrease the prevalence of children requiring surgical intervention [17]. In addition, rigorous assessment of radiographers and practices is required, which has made this infeasible in the vast and diverse health environment of Australia. Evidence for selective hip screening remains controversial, although it is common practice in Australia. A randomised controlled trial by Rosendahl et al. in 1994 found no statistical difference between late diagnosis of DDH when comparing ultrasound screening and clinical screening in the presence of risk factors, to clinical screening only (universal or selective) [18]. Two authors from the 1994 study later published in 2014 supporting the use of selective ultrasound screening for DDH [19]. The American Academy of Orthopaedic Surgeons Clinical Practice Guidelines (2022) support imaging before 6 months of age in children with one or more risk factors for DDH [20]. Despite the controversy, should supplemental selective ultrasound screening be used as part of practice, it is important to correctly identify risk factors for DDH.

A limitation of this study is the comparison to interstate registries. While all included participants in our dataset received an ultrasound at 6–8 weeks of age, in the WA and SA populations ultrasounds would have been performed based on clinical and selective screening. This may have underestimated the prevalence of DDH in these comparison populations. This underestimation is mitigated by the inclusion of late‐diagnosis DDH in the WA and SA datasets. The incidence of late‐diagnosed DDH in the WA dataset is 0.49 per 1000 [13]. This is consistent with a 2023 systematic review by Choek et al., with a determined incidence of late diagnosis DDH using selective screening of 0.45 (95% CI 0.31–0.61) per 1000 [17].

Differences between the study population and comparison population also include interstate variation in ethnogeographic ancestry. There are marked differences in international prevalence rates of DDH, based on ancestral backgrounds. Australian census data from 2016 suggests there is some variation of ancestral backgrounds between NSW, WA and SA populations [21]. Included participants would predominantly be from metropolitan Sydney, with regional children managed locally. It is impossible to quantify the direction and size of bias introduced by uncertainty due to state and regional variation. Another difference is an increased proportion of males in our study population, compared to the population datasets, which potentially underestimates the relationship between CTEV and DDH, as DDH is more common in females [1].

We can also observe the prevalence of DDH in our study population differs from a 2023 systematic review by Tao et al. of 14.0 (95% CI 8.6–22.8) per 1000 in the general population [22]. This study included almost 3.5 million infants. While the precise statistical relationship between CTEV and DDH in our study is impacted by the limitation of the dataset comparisons, the strength of the findings suggests that there is a relationship.

The effect of potential treatment and confirmation bias in this cohort is difficult to assess. Traditional teaching is that CTEV is not related to DDH, and yet the longstanding practice at SCH was to instigate ultrasound analysis. This practice was asynchronous to published scientific data at that time and triggered this audit of our dataset. A positive ultrasound result may bias treatment decision making and falsely increase the DDH treatment cohort.

Further bias could have been introduced through excluding 23 subjects with missing data from the primary analysis. A secondary analysis was performed to account for this bias, providing a conservative estimate of the correlation between CTEV and DDH. The prevalence rates remained higher than the general population, and the relative risk was still substantially greater than the general population.

Genetics are a significant cause of CTEV, with individual genes identified [7]. Current understanding indicates the pathogenesis of DDH has both environmental and genetic contributions [23]. Individual genes causing DDH have not been identified, but the strong familial link between first‐degree relatives [16] is driving continued genetic research. The correlation between CTEV and DDH may provide further opportunities to research the genes associated with each condition, to better understand the relationship.

With postnatal traditional swaddling a risk factor for DDH [3], there is some suggestion from case studies that hip dysplasia can develop due to Ponseti casting [24]. Larger cohort studies would be required to confirm this relationship.

This study demonstrates an increased prevalence of DDH in a population of infants with idiopathic CTEV. The relative risk of DDH in the study population is five to 10 times greater than whole population cohorts from WA and SA, respectively. The relative risks are comparable to other known risk factors for DDH, including breech presentation and positive family history. When selective hip ultrasound screening is used, we believe idiopathic CTEV should be considered a risk factor for DDH.

## Ethics Statement

Ethics approval was obtained from the Sydney Children's Hospitals Network Human Research Ethics Committee 2020/ETH01509.

## Conflicts of Interest

The authors declare no conflicts of interest.

## Supporting information


**Data S1.**Supporting Information.
