# Supplementary material for: The Relationship Between Developmental Dysplasia of the Hip (DDH) and Congenital Talipes Equinovarus (CTEV)—A Retrospective Case Series
Source: J Paediatr Child Health. 2025 May 19;61(7):1116–21. doi: 10.1111/jpc.70089 (PMC12211549; doi:10.1111/jpc.70089)
Supplement: Supplementary file 1 — Data S1.Supporting Information. [file JPC-61-1116-s001.docx]

**Supporting Information**

| Subject | Sex | CTEV | DDH | Treatment | Torticollis | Breech |
| --- | --- | --- | --- | --- | --- | --- |
| 1 | F | R | L | Brace | N | N |
| 2 | M | B | B | Brace | U | N |
| 3 | F | R | R | Brace | N | N |
| 4 | F | R | L | Brace | N | N |
| 5 | F | B | L | Brace | N | N |
| 6 | M | L | L | Brace | U | N |
| 7 | M | B | B | Brace | N | N |
| 8 | F | B | B | Brace | N | N |
| 9 | M | L | L | Surgery | N | N |
| 10 | F | B | B | Brace | N | N |
| 11 | M | L | L | Brace | N | N |
| 12 | M | R | L | Brace | N | N |

**Table S1:** Details of Subjects with DDH. F female M male. L left R right B bilateral. N no U unknown (missing data).
